# Metabolomic analysis of the human placenta reveals perturbations in amino acids, purine metabolites, and small organic acids in spontaneous preterm birth

**DOI:** 10.17179/excli2023-6785

**Published:** 2024-02-13

**Authors:** Eva Cifkova, Rona Karahoda, Jaroslav Stranik, Cilia Abad, Marian Kacerovsky, Miroslav Lisa, Frantisek Staud

**Affiliations:** 1 Department of Chemistry, Faculty of Science, University of Hradec Kralove, Rokitanskeho 62, 50003, Hradec Kralove, Czech Republic; 2 Department of Pharmacology and Toxicology, Faculty of Pharmacy in Hradec Kralove, Charles University, Akademika Heyrovskeho 1203/8, 50005, Hradec Kralove, Czech Republic; 3 Department of Obstetrics and Gynecology, University Hospital Hradec Kralove, Sokolska 581, 50005, Hradec Kralove, Czech Republic

**Keywords:** placenta, preterm birth, metabolomics, inflammation, metabolism

## Abstract

Spontaneous preterm delivery presents one of the most complex challenges in obstetrics and is a leading cause of perinatal morbidity and mortality. Although it is a common endpoint for multiple pathological processes, the mechanisms governing the etiological complexity of spontaneous preterm birth and the placental responses are poorly understood. This study examined placental tissues collected between May 2019 and May 2022 from a well-defined cohort of women who experienced spontaneous preterm birth (n = 72) and healthy full-term deliveries (n = 30). Placental metabolomic profiling of polar metabolites was performed using Ultra-High Performance Liquid Chromatography/Mass Spectrometry (UHPLC/MS) analysis. The resulting data were analyzed using multi- and univariate statistical methods followed by unsupervised clustering. A comprehensive metabolomic evaluation of the placenta revealed that spontaneous preterm birth was associated with significant changes in the levels of 34 polar metabolites involved in intracellular energy metabolism and biochemical activity, including amino acids, purine metabolites, and small organic acids. We found that neither the preterm delivery phenotype nor the inflammatory response explain the reported differential placental metabolome. However, unsupervised clustering revealed two molecular subtypes of placentas from spontaneous preterm pregnancies exhibiting differential enrichment of clinical parameters. We also identified differences between early and late preterm samples, suggesting distinct placental functions in early spontaneous preterm delivery. Altogether, we present evidence that spontaneous preterm birth is associated with significant changes in the level of placental polar metabolites. Dysregulation of the placental metabolome may underpin important (patho)physiological mechanisms involved in preterm birth etiology and long-term neonatal outcomes.

## Introduction

Preterm delivery, defined as the onset of labor before 37 weeks of gestation, presents one of the most complex challenges in obstetrics. The majority of preterm births are spontaneous (non-medically-indicated) following premature rupture of the membranes (PPROM) or result from preterm labor with intact membranes (PTL) (Goldenberg et al., 2008[22]). Despite advances in clinical practice, premature birth remains a globally relevant problem and a leading cause of perinatal morbidity and mortality (Blencowe et al., 2012[7]). Research has shown that delivery timing significantly influences the neonatal complications associated with spontaneous preterm birth (ACOG, 2013[2]). While early preterm infants (< 34 weeks) exhibit a higher incidence of mortality and morbidity, late preterm newborns (34 - 37 weeks) have a significantly increased risk of developing health complications compared to those born at term (Saigal and Doyle, 2008[51]; Teune et al., 2011[60]). Because it causes long-term disabilities that create a significant economic burden, spontaneous preterm birth has an enormous impact on the healthcare system, and infants born preterm present a challenge within the field of perinatology (Ward and Beachy, 2003[64]).

Spontaneous preterm birth can be seen as a multifactorial disorder that is a common endpoint of multiple pathological processes. However, the mechanisms contributing to this etiological complexity and the long-term effects of spontaneous preterm birth are poorly understood (Goldenberg et al., 2008[22]). Consequently, studies have sought to clarify the placenta's activity as the core regulator of the intrauterine environment (Jaiman et al., 2022[27]). A temporary organ at the interface between the mother and fetus, the placenta acts in several ways to create and maintain optimal in-utero conditions for fetal development (Staud and Karahoda, 2018[57]). It is also an endocrine organ that synthesizes organic compounds, hormones, neurotransmitters, and vasoactive factors that are essential for placental development and growth but may also be secreted into the fetal circulation, affecting fetal development and programming (Staud and Karahoda, 2018[57]). Accumulating evidence suggests that placental insufficiency, altered placental vascularization, and maturational defects of placental villi may contribute to spontaneous preterm birth (Arias et al., 1993[5]; Jaiman et al., 2021[28], 2022[27]; Morgan, 2014[42]). Moreover, the placenta is continuously exposed to the effects of hormones and cytokines in maternal circulation because of its unique position (Hsiao and Patterson, 2011[25]). We and others have shown that the adverse conditions associated with infection/inflammation-related preterm delivery can profoundly affect the placenta's structure and endocrine/transport functions (Hsiao and Patterson, 2011[25]; Karahoda et al., 2021[31]), and may also influence the postnatal offspring phenotype (Sferruzzi-Perri and Camm, 2016[56]). The cellular processes underlying placental functions must therefore be understood to clarify the pathological complexity contributing to spontaneous preterm birth and its long-term effects on fetal development.

Metabolomics employs cutting-edge technologies to detect and quantify metabolic changes caused by pathophysiological processes in specific organ systems. It is arguably a more powerful tool than transcriptomics and proteomics because metabolites are the end products of gene and protein expression and thus directly reflect the cellular state at the functional level (Johnson et al., 2016[30]). Studies on metabolites involved in important placental biological processes can therefore provide critical insights into the pathological processes associated with spontaneous preterm delivery (Romero et al., 2010[50]). While “omics” techniques have provided deep insights into the differential metabolome profiles of whole blood, amniotic fluid, urine, and the vagina (Carter et al., 2019[14]; Jehan et al., 2020[29]; Kindschuh et al., 2023[32]; Romero et al., 2010[50]), few studies have examined metabolic changes in placentas from pregnancies affected by spontaneous preterm birth (Elshenawy et al., 2020[18]). This study therefore characterized the placental metabolome in a clinical cohort (n = 102) comprising spontaneous early preterm and late preterm births as well as healthy term pregnancies. We focused on polar metabolites with the aim of identifying significant differences in metabolite levels/ratios between healthy and preterm placentas as well as dysregulated biochemical pathways in the latter. In addition, we investigated potential mechanistic links between the placental metabolome, gestational age at delivery, and maternal and intra-amniotic markers of inflammation such as maternal serum C-reactive protein (CRP) and white blood cell (WBC) count, and amniotic fluid interleukin-6 (IL-6) in the preterm group. We hypothesized that changes in the placental metabolome underpin important pathophysiological processes involved in spontaneous preterm birth and long-term neonatal outcomes. 

## Methods

### Clinical diagnosis and treatment

Gestational age was established by first-trimester fetal biometry. PTL diagnosis was based on regular uterine contractions and cervical length, measured as previously described (Stranik et al., 2022[58]; van Baaren et al., 2014[62]). PPROM was distinguished by the pooling of amniotic fluid in the posterior fornix of the vagina. In cases of uncertain PPROM diagnosis, vaginal fluid was analyzed for the presence of insulin-like growth factor-binding proteins, as described previously (Karahoda et al., 2021[31]). 

To investigate the intra-amniotic environment, ultrasound-guided transabdominal amniocentesis was performed on admission. Amniotic fluid IL-6 concentrations were measured using an automated electrochemiluminescence immunoassay (Roche Diagnostics, Basel, Switzerland), and intraamniotic infection (IAI) was diagnosed based on an IL-6 concentration ≥ 3 ng/mL in the amniotic fluid (Musilova et al., 2020[43]). Microbial invasion of the amniotic cavity (MIAC) was diagnosed based on a positive PCR analysis for any combination of *Ureaplasma* species, *Mycoplasma hominis*, and *Chlamydia trachomatis*, positivity for the 16S rRNA gene, aerobic/anaerobic cultivation of the amniotic fluid, or some combination of these parameters. 

For standard histological examination, tissue-block sections of the placenta, umbilical cord, and fetal membranes were placed in paraffin after delivery and stained with hematoxylin and eosin. Acute histological chorioamnionitis (HCA) was diagnosed based on the presence of histological grades of chorion-decidua 3 - 4, chorionic plate 3 - 4, umbilical cord 1 - 4, and/or amnion 1 - 4 (Salafia et al., 1989[52]), as previously described (Karahoda et al., 2021[31]). The nature and intensity of the placental inflammatory response was classified as maternal or fetal using the following scheme based on HCA grades: maternal (presence of histological grades of chorion-decidua 3 - 4 and/or chorionic plate 3 and/or amnion 1 - 4) or fetal (presence of histological grades of chorionic plate 4 and/or umbilical cord 1 - 4).

Maternal blood samples were collected at the time of delivery by venipuncture of the cubital vein. WBC counts and CRP levels were determined immediately after sampling. 

The patients received corticosteroids, antibiotics, or tocolytics, depending on their diagnosis. Specifically, PTL diagnosis was managed with a course of betamethasone and tocolytic therapy (intravenous atosiban, or nifedipine administered orally for 48 hours). When intra-amniotic inflammation was present, patients were treated with intravenous clarithromycin for seven days unless delivery occurred earlier. Women with PTL who were positive for group B Streptococcus received intravenous benzylpenicillin (clindamycin, in case of penicillin allergy) during active labor. 

Women with PPROM were treated with antibiotics. When no intra-amniotic inflammation was present, the patients received benzylpenicillin (or clindamycin). In cases of intra-amniotic inflammation, intravenous clarithromycin was administered for seven days; the antibiotic treatment was modified if microbial invasion of the amniotic cavity was detected. When PPROM was diagnosed at gestational age < 35 weeks, the women also received corticosteroids (betamethasone) to accelerate fetal lung maturation and reduce neonatal mortality and morbidity. Women with PPROM were managed expectantly except for those with intra-amniotic infection and gestational age > 28 weeks, for whom labor was induced or an elective cesarean section was performed within 72 h of admission (Stranik et al., 2021[59]).

### Placental collection

Tissue collection occurred between May 2019 and May 2022 at the Department of Obstetrics and Gynecology, University Hospital in Hradec Kralove, Czech Republic. Placental villous tissue (approximately a 5 cm^2^ piece) was collected immediately after delivery and stored at −80 °C until further processing. The study examined a well-characterized clinical cohort of healthy term pregnancies (n = 30) and spontaneous preterm deliveries (n = 72). Individuals with preeclampsia, intrauterine growth restriction (IUGR), diabetes mellitus, gestational diabetes mellitus, and gestational hypertension, as well as complications such as structural malformations or chromosomal abnormalities of the fetus, fetal growth restriction, vaginal bleeding, and/or signs of fetal hypoxia were excluded from the study. Table 1(Tab. 1) shows the demographic and clinical characteristics of the pregnant women enrolled in the study. The differences between the PTL (n = 22) and PPROM (n = 50) pregnancies are summarized in Supplementary Table 1. 

All research activities were conducted in accordance with the ethical standards established in the Declaration of Helsinki and were approved by the Research Ethics Committee of the University Hospital in Hradec Kralove, Czech Republic (Approval No. 201006 S15P). Written informed consent was obtained from all patients.

### Sample preparation

The placenta was manually dissected and cleared of chorionic plate and maternal decidua. Approximately 100 - 200 mg of villous tissue was washed twice with ice-cold 0.9 % sodium chloride to remove excess blood and homogenized with methanol (8 µL/mg of tissue) (LC/MS grade, Honeywell, Charlotte, North Carolina, USA), 1.2-1.4 mm zirconium oxide beads yttrium stabilized (2.5 mg/mg of tissue) (SiLibeads, Warmensteinach, Germany), and Garnet matrix (3 mg/mg of tissue) (MP Biomedicals, Santa Ana, California, USA) using FastPrep-24^TM^ 5G (MP Biomedicals) in 3 cycles of 30 s each with 30 s pauses on an ice bath at a device speed of 6 m/s. The resulting tissue homogenate (200 µL) was mixed with 2.5 µL of mixed internal standards (ISs; Supplementary Table 2), 1320 µL of methanol, and 380 µL of 1 % formic acid (LC/MS grade, Honeywell) in water (LC/MS grade, Merck, Kenilworth, New Jersey, USA). This mixture was vortexed for 10 min and centrifuged for 5 min at 16,000 rpm. Then, 1520 µL of supernatant was collected, evaporated with a vacuum concentrator (Eppendorf, Hamburg, Germany), and reconstituted in 25 µL of mobile phase A (0.1 % formic acid in water) before UHPLC/MS analysis. A quality control (QC) sample for UHPLC/MS analyses was prepared by pooling 10 µL of sample homogenates, spiking with ISs, and performing extraction using the same procedure as for samples. Prepared QC extracts were mixed and divided into 25 µL aliquots. All samples were strictly randomized, and QC aliquots were injected after every 10^th^ experimental sample.

### UHPLC/MS conditions

UHPLC/MS metabolomic analysis of placenta samples was performed with an Acquity I-class UPLC instrument and a Vion IMS QTOF mass spectrometer (Waters, Milford, MA, USA) using an Acquity UPLC HSS T3 column (150 × 2.1 mm, 1.8 µm, Waters) with a flow rate of 0.3 mL/min, an injection volume of 3 µL, an autosampler temperature of 8 °C, a column temperature of 30 °C and the following mobile phase gradient: 0 min - 100 % A, 2 min - 100 % A, 8 min - 100 % B, 10 min - 100 % B, 11 min - 100 % A, 20 min - 100 % A, where A was 0.1 % formic acid in water and B was 0.1 % formic acid in methanol. Electrospray ionization (ESI) full-scan mass spectra were acquired in positive-ion mode with the following tuning parameters: mass range 50-1000, soft transition mode, scan time 0.2 s, capillary voltage 0.5 kV, cone voltage 10 V, source offset 50 V, source temperature 130 °C, desolvation temperature 600 °C, cone gas flow 50 L/h, and desolvation gas flow 800 L/h. Leucine enkephaline was used as the lock mass for all experiments. Method validation based on selectivity, accuracy, precision, calibration curves, detection and quantification limits, matrix effects, extraction efficiency, and carryover (Supplementary Table 3) was performed as described previously (Lísa et al., 2017[36]).

### Data processing and statistical analysis

Metabolites were identified in the pooled placenta samples based on accurate m/z measurements, retention times of identical standards, and characteristic fragments in tandem mass spectra acquired in ion mobility mode. Deuterated standards of selected metabolites added to samples during sample preparation were used as ISs to validate the UHPLC/MS method and enable quality assurance of the metabolomic analysis by measurement of extraction efficiencies, retention times, and signal stability. The raw data from the metabolomic analysis were processed using Unifi software (Waters) with a mass tolerance of 10 mDa. For statistical analysis, metabolite signals were corrected using the QC sample by examining the ratio of the peak area for each metabolite in the sample to that of the same metabolite in the closest QC sample (*i.e*., A_sample_/A_QC_). Multivariate statistical analyses including principal component analysis (PCA) and orthogonal partial least squares discriminant analysis (OPLS-DA) were performed using the SIMCA software package (version 13.0, Umetrics, Umeå, Sweden). Univariate analyses such as box plots were conducted and visualized using GraphPad Prism (version 9.0, San Diego, California, USA). Data were preprocessed before multivariate statistical evaluation using Pareto scaling, mean centering, and logarithm transformation. P-values were calculated with GraphPad Prism using the nonparametric Mann-Whitney test or Fisher's exact test where appropriate, and then the Bonferroni correction (α = 0.05/number of metabolites) and Benjamini-Hochberg correction (α = 0.05*ranking by p-value/number of metabolites) were applied (Supplementary Table 4). Fold changes were calculated as the ratio of the difference between the medians of two groups to the median of the control group. The Cytoscape program (https://cytoscape.org) was used to visualize differences between the preterm and term placentas. In these visualizations, the sizes of the circles indicate p-values and the color scale indicates the fold change. Metabolic pathway analysis was performed using MetaboAnalyst 5.0 (https://www.metaboanalyst.ca/), where metabolic pathways are represented as circles based on their p-values and topological properties (pathway impact); redder colors indicate more significant changes (i.e., higher p-values), while larger circles correspond to higher impact scores. Finally, bubble plots were generated in GraphPad Prism. In these plots, the node color gradient indicates the fold change or Spearman's correlation coefficient r, while the node radius indicates the negative log of the p-value (see Supplementary Table 5 for correlation analysis results).

## Results

### Clinical and demographic characteristics of the cohort

The demographic and clinical characteristics of the pregnant women participating in the study are presented in Table 1(Tab. 1). In total, 102 placentas were analyzed, of which 72 were spontaneously delivered preterm (39 early and 33 late), while 30 underwent healthy term delivery. Medically-induced preterm deliveries and other pregnancy complications were excluded from the study. Birth weight and gestational age at delivery differed significantly between spontaneous preterm and term births (p < 0.001). While there was no difference in pre-pregnancy BMI between the two groups, BMI at admission was significantly higher for the term group than the spontaneous preterm group (p = 0.0172). No significant between-group differences in fetal sex distribution were detected. However, whereas spontaneous preterm birth was primarily associated with vaginal delivery, term pregnancies were predominantly delivered by Caesarian section (p = 0.0019). Furthermore, tocolytics, antibiotics, and corticosteroids were routinely used in preterm deliveries.

Within the spontaneous preterm group, 22 presented as PTL, and 50 were diagnosed with PPROM (Supplementary Table 1). PPROM was linked to increased gestational age at delivery (p = 0.0082) and newborn weight (p = 0.0061). Tocolytics were more likely to be administered in PTL deliveries (p = 0.0004), which were also accompanied by higher amniotic fluid IL-6 concentrations at admission (p = 0.0018). The PTL and PPROM groups did not differ with respect to the incidence of MIAC, HCA, funisitis, male fetal sex, caesarian delivery, parity, or treatment with corticosteroids or antibiotics. There were also no significant differences in maternal serum CRP levels or the WBC count at delivery. 

### Placental metabolomic profile in spontaneous preterm birth and healthy term pregnancy

**Figure 1:**
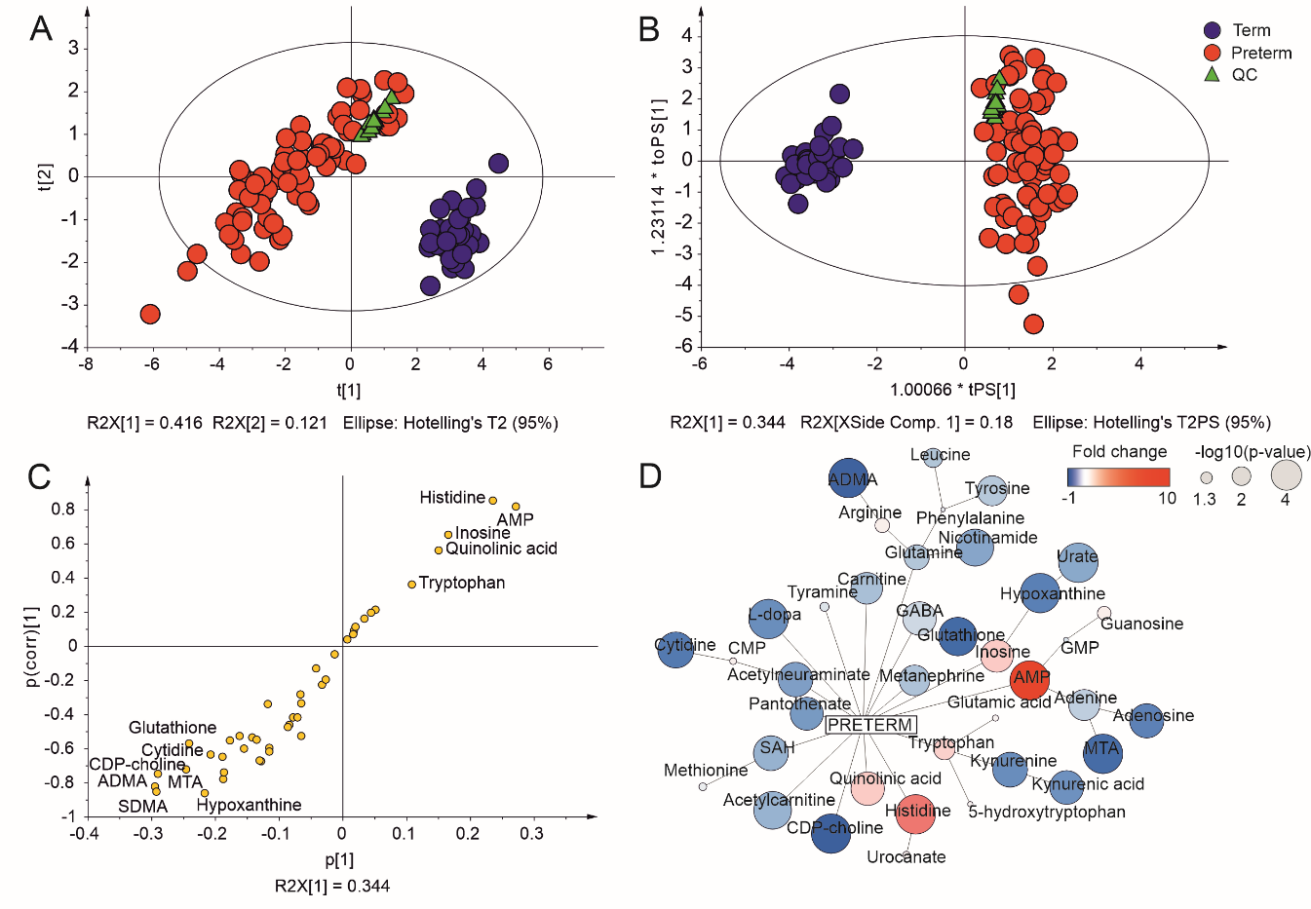
Biostatistical analysis of metabolomic profiles in spontaneous preterm and term placenta. (A) Principal component analysis score plot. (B) Orthogonal partial least squares (OPLS) score plot. (C) OPLS S plot. (D) Network visualization of interactions between 43 metabolites and their regulation in spontaneous preterm birth. The size of the circles represents the significance of the difference between metabolite levels in spontaneous preterm and healthy term placentas (based on p-values), while the color indicates the fold change in metabolite levels when comparing preterm and term placentas (blue - downregulated, red - upregulated).

A total of 43 polar metabolites were identified by UHPLC/MS untargeted analysis of the pooled placenta samples (see Supplementary Table 4 for the complete list of metabolites identified). Each metabolite was identified based on ESI mass spectra with high resolving power and m/z accuracy in conjunction with characteristic fragmentation behavior in tandem mass spectra in ion mobility mode, and identifications were confirmed by comparison to analytical standards where possible. The levels of identified metabolites in the placenta samples were then determined and a multivariate data analysis using unsupervised PCA was performed to compare the metabolomic profiles of placenta samples from the whole clinical cohort. The term and preterm delivery groups are clearly separated within the space spanned by the PCA model's first two components (Figure 1A(Fig. 1)), which explained 41.6 and 12.1 % of the variance in the data, respectively. A supervised OPLS analysis was then performed to clarify the influence of individual metabolites on the clustering of the term and preterm placentas. The resulting scores' plot (Figure 1B(Fig. 1)) shows the variability between and within classes on the horizontal and vertical axes, respectively, and once again demonstrates clear separation of the preterm and term placentas. Importantly, the QC sample was regularly injected throughout the study to confirm that the performance of the analytical apparatus remained consistent, and the tightness of the metabolic profiles in the PCA/OPLS plot demonstrates the reliability of the metabolomic results. The OPLS S plot (Figure 1C(Fig. 1)) shows identified metabolites whose abundance in the preterm placenta was significantly higher (n = 12) or lower (n = 31) than in term placentas. These dysregulated metabolites were then visualized by generating a network interaction map in Cytoscape (Figure 1D(Fig. 1)) based on univariate statistical analyses to clarify the functional consequences of their dysregulation and the relationships between them. 

We next investigated potential correlations between the spontaneous preterm birth phenotypes (PPROM vs. PTL) and the placental metabolome. In addition, since over 90 % of spontaneous preterm pregnancies were complicated by HCA, we stratified the population based on the HCA inflammatory response (none vs. maternal vs. fetal). However, a PCA of the metabolomic data for these placental subgroups revealed no correlations between the placental metabolome and spontaneous preterm phenotype (Supplementary Figure 1A) or HCA grades (Supplementary Figure 1B).

### Differential metabolite analysis

**Figure 2:**
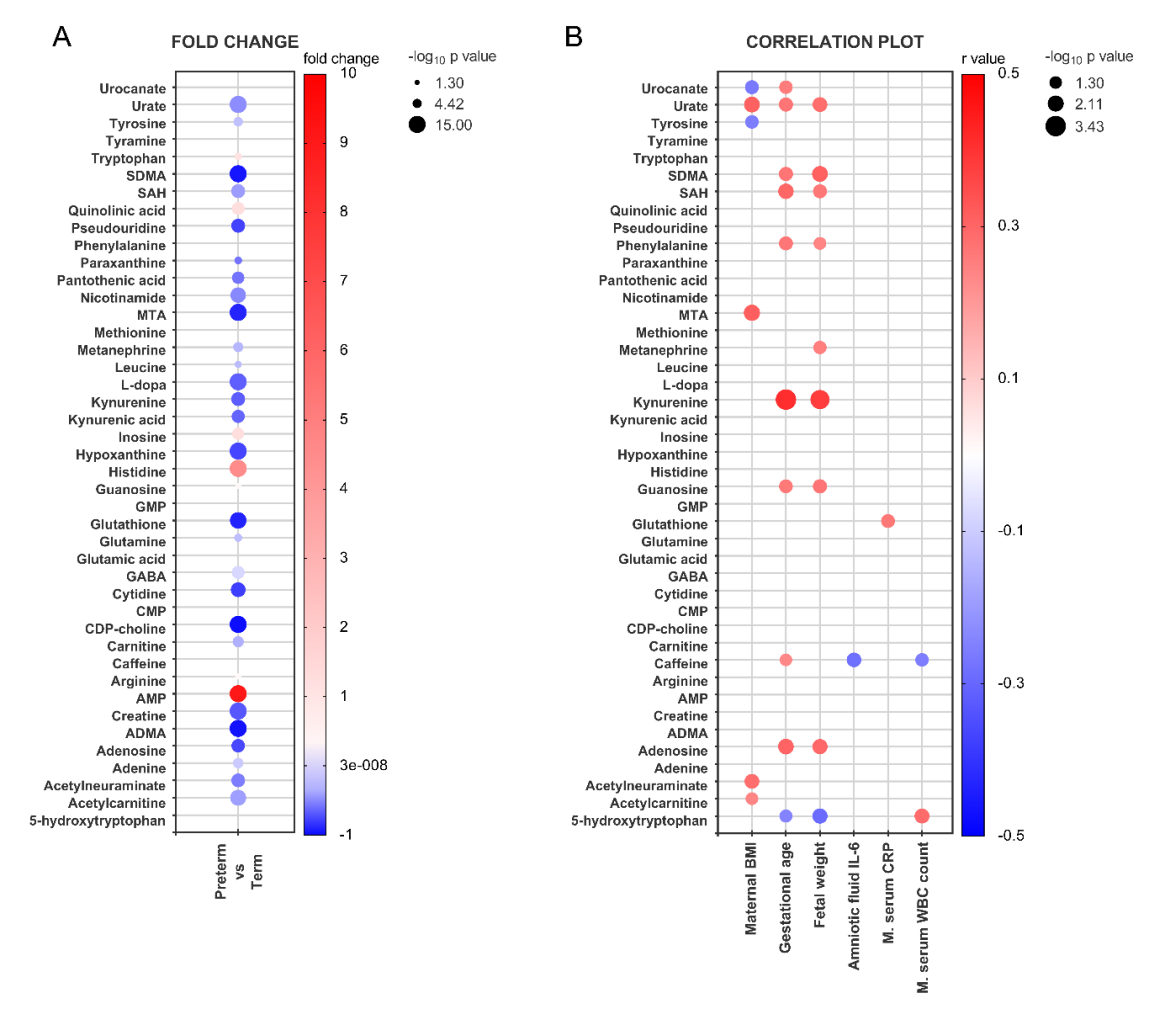
Changes in the placental metabolomic profile in spontaneous preterm birth. (A) Bubble plot highlighting metabolites that are dysregulated in preterm placentas when compared to term deliveries. The node color gradient indicates the difference in metabolite levels between term and preterm placentas, expressed as a fold change, while the node radius reflects the negative log of the p-value. (B) Correlations between clinical patient data and individual metabolite levels in spontaneous preterm birth placentas. The node color gradient indicates the strength of the correlation based on Spearman's correlation coefficient r, while the node radius reflects the negative log of the p-value. The bubbles are limited to metabolites with negative log p-values greater than 1.3, indicating only significant correlations. The raw data can be found in Supplementary Tables 4 and 5.

A differential metabolite analysis comparing spontaneous preterm and term placentas revealed 34 polar metabolites whose levels differed significantly between the two groups (Figure 2A(Fig. 2)). To elucidate the relationship between individual metabolites and spontaneous preterm birth, we next performed a correlation analysis focusing on maternal BMI at admission, gestational age at delivery, newborn weight, and maternal and intra-amniotic markers of inflammation. The resulting bubble plot is shown in Figure 2B(Fig. 2). The quantitative correlations between metabolite levels and the targeted factors were generally moderate and positive (Spearman's correlation: ≤ 0.41); gestational age at delivery and newborn weight exhibited both the most and the strongest correlations. 

To clarify the biological implications of metabolic dysregulation in spontaneous preterm placentas, we performed a metabolic pathway analysis using the KEGG database (Supplementary Figure 2). The dysregulated metabolites were enriched in 28 metabolic pathways. Pathway enrichment analysis indicated that the 10 most enriched altered pathways were purine metabolism, beta-alanine metabolism, aminoacyl-tRNA biosynthesis, histidine metabolism, arginine and proline metabolism, glycine, serine and threonine metabolism, glycerophospholipid metabolism, cysteine and methionine metabolism, tyrosine metabolism, and nicotinate and nicotinamide metabolism. Furthermore, a pathway topology analysis identified phenylalanine, tyrosine, and tryptophan biosynthesis as the most important pathway involving tyrosine and phenylalanine.

### Clinical analysis of the placental metabolomic clusters in spontaneous preterm birth

**Figure 3:**
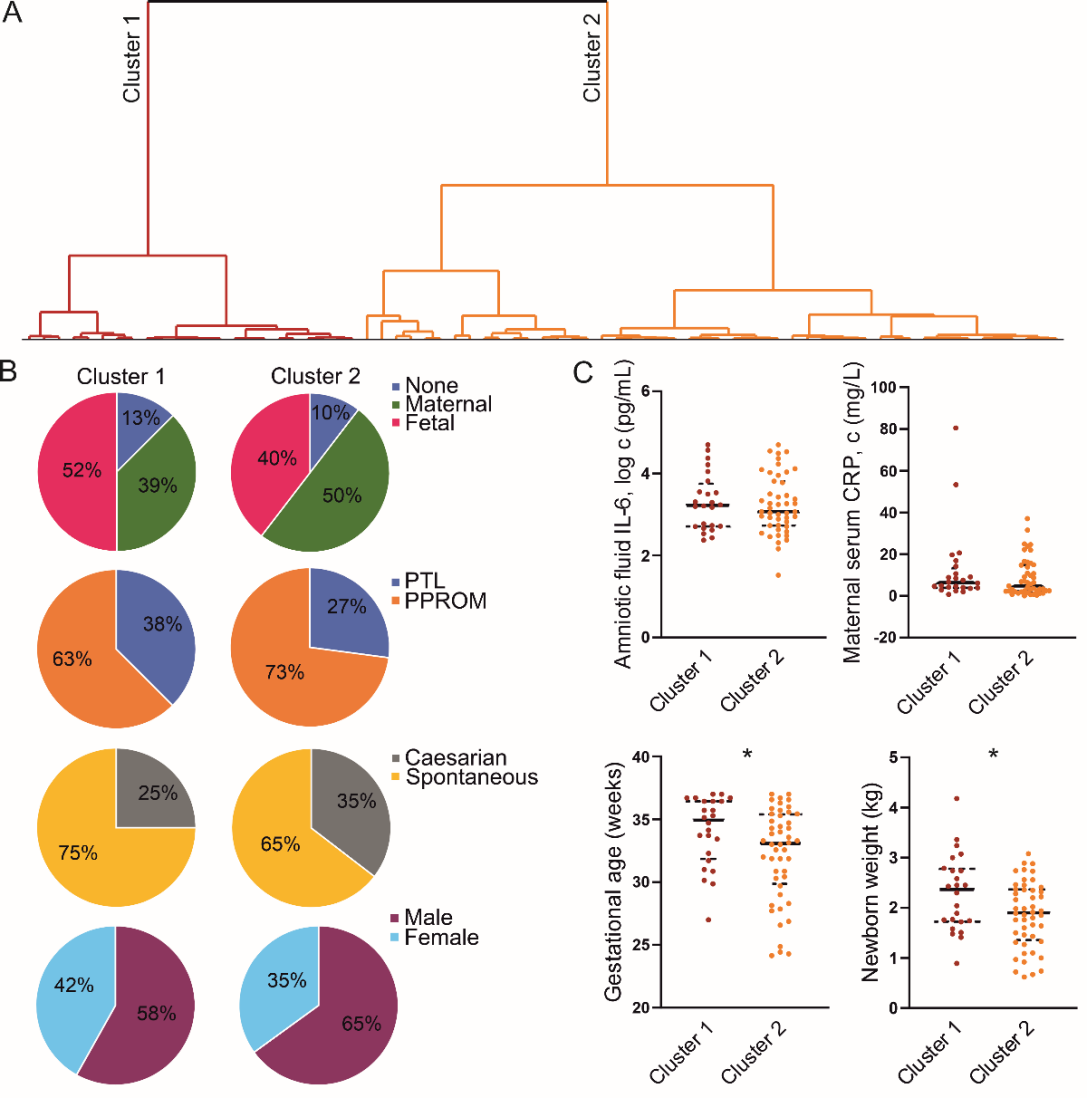
Unsupervised clustering of spontaneous preterm pregnancies based on the placental metabolomic signature. (A) Analysis of 72 placentas revealed two main clusters: Cluster 1, represented in red (n = 23), and Cluster 2 in yellow (n = 49). (B) Distributions of HCA inflammatory responses, preterm birth phenotype, delivery mode, and fetal sex in Clusters 1 and 2. (C) Differences in maternal and intra-amniotic markers of inflammation, gestational age at delivery, and newborn weight between Clusters 1 and 2. Statistical analyses were performed using the nonparametric Mann-Whitney test.

The metabolomic data were subjected to hierarchical clustering to characterize the relationships between phenotypic factors and metabolites. Unsupervised clustering of the 72 placentas (Figure 3A(Fig. 3)) based on their metabolomic profiles separated them into two clusters with sizes of 23 (Cluster 1) and 49 (Cluster 2). These clusters did not differ significantly in HCA grade distribution, preterm phenotype (PPROM vs. PTL), delivery mode, or fetal sex (Figure 3B(Fig. 3)). In addition, the clusters had similar levels of amniotic fluid IL-6 and maternal serum CRP (Figure 3C(Fig. 3)). However, placentas from Cluster 2 were delivered significantly earlier than those from Cluster 1 (p = 0.034), so the newborn weight was significantly lower in these pregnancies (Figure 3C(Fig. 3)). 

**Figure 4:**
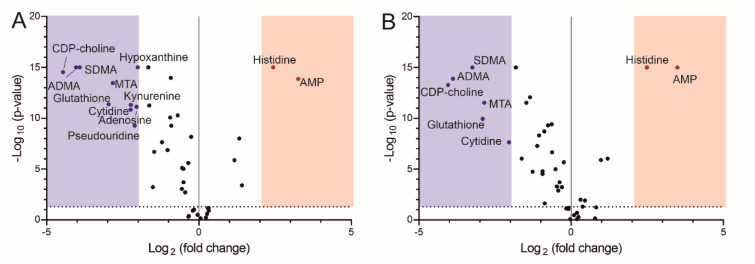
Volcano plots showing metabolites exhibiting pronounced and significant changes when comparing (A) early preterm and (B) late preterm placentas to healthy term placentas. Raw data can be found in Supplementary Table 4.

Since the clustering based on metabolomic profile correlated with the gestational age at delivery, we next analyzed the gestational age subgroups included in the study (early and late preterm) separately, using healthy full-term pregnancies as a reference group. This revealed that the number of dysregulated metabolites identified by comparison to healthy term samples was higher in early preterm placentas (Figure 4A(Fig. 4)) than in the late preterm group (Figure 4B(Fig. 4)). Box plots for individual metabolites are shown in Supplementary Figure 3. Overall, our metabolomic analysis showed that both the magnitude and the significance of metabolite dysregulation were greater in early preterm placentas than in those from late preterm births. 

### Identification of metabolic disturbances in spontaneous preterm birth based on placental product/substrate ratios 

**Figure 5:**
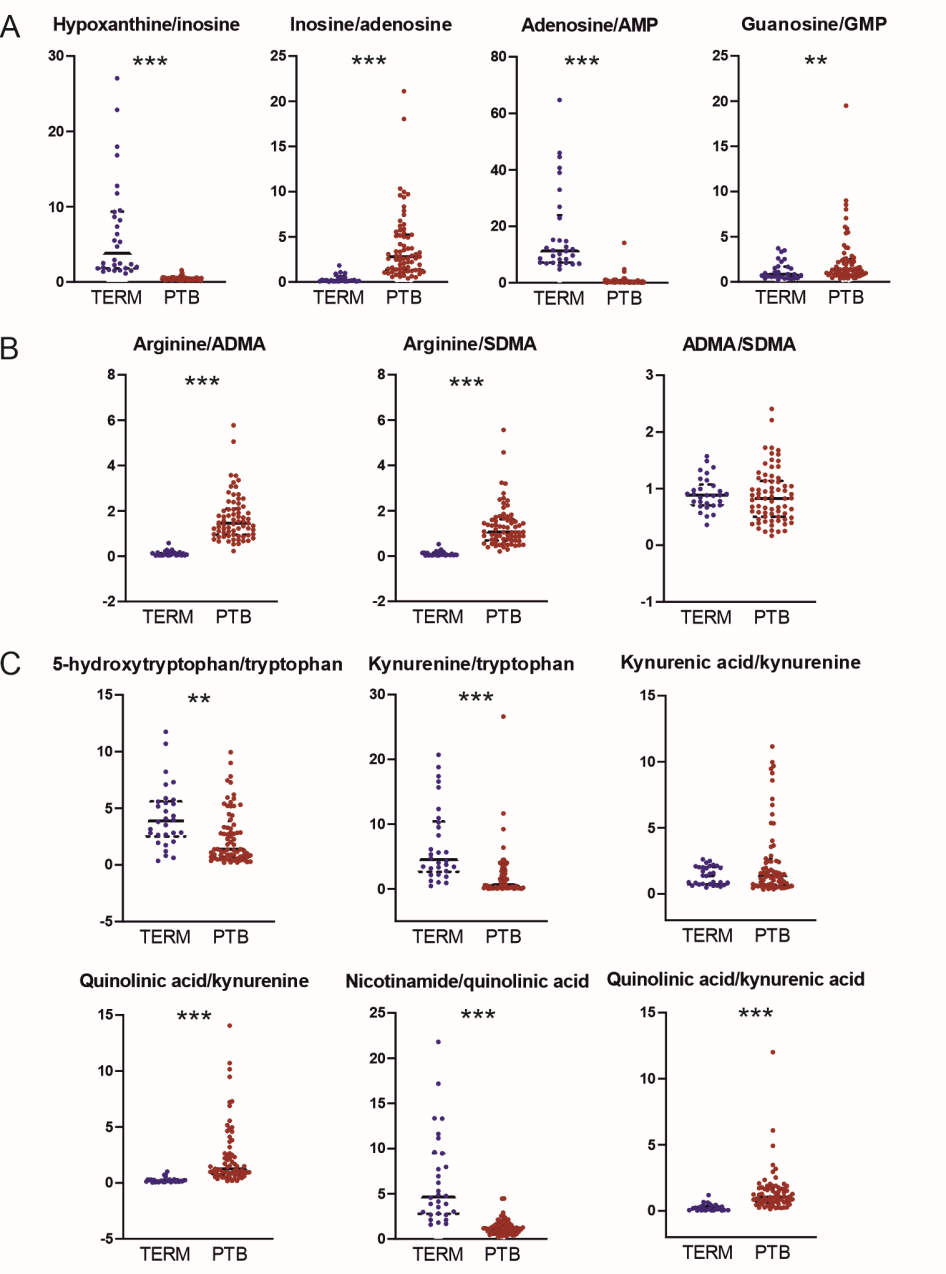
Placental product/substrate ratios as a proxy for metabolic disturbances in spontaneous preterm birth. Box plots of differences between term and spontaneous preterm (PTB) placentas with respect to metabolite ratios for purine (A), arginine (B), and tryptophan (C) metabolism. Statistical analysis was performed using the nonparametric Mann-Whitney test.

Because metabolites are products or substrates of metabolic enzymes, their tissue levels reflect the activity of those enzymes. Analysis of product/substrate ratios can therefore provide valuable information on the (patho)physiological state of tissues. After considering the set of polar metabolites identified in the placenta, we focused our analysis on three metabolic pathways exhibiting broad product-substrate relationships: purine metabolism (Figure 5A(Fig. 5)), arginine metabolism (Figure 5B(Fig. 5)), and tryptophan pathway (Figure 5C(Fig. 5)).

For purine metabolites, the adenosine/ AMP and hypoxanthine/inosine ratios were significantly lower in preterm placentas than in term placentas, while the inosine/adenosine and guanosine/GMP ratios were significantly higher (Figure 5A(Fig. 5)). For arginine and its methylated metabolites, preterm placentas exhibited significantly elevated arginine/ADMA and arginine/SDMA ratios, but the ADMA/ SDMA ratio was similar in term and preterm placentas (Figure 5B(Fig. 5)). Preterm placentas also had significantly lower 5-hydroxytryptophan/tryptophan and kynurenine/tryptophan ratios than term placentas, indicating significantly reduced fluxes of tryptophan along the serotonin and kynurenine pathways (Figure 5C(Fig. 5)). The available kynurenine in the preterm placenta appeared to be preferentially converted into quinolinic acid because the preterm placentas had an elevated quinolinic acid/kynurenine ratio and a significantly elevated quinolinic acid/kynurenic acid ratio. Finally, the preterm placentas had a reduced nicotinamide/quinolinic acid ratio (Figure 5C(Fig. 5)). However, it should be noted that each of the metabolites mentioned above is a product and/or substrate of multiple reactions and their *in vivo *concentrations may be affected by multiple modulating factors (Badawy and Guillemin, 2019[6]). Therefore, these results should be interpreted with care when trying to identify (patho)physiological signatures characteristic of the preterm placenta. 

## Discussion

Using UHPLC/MS and robust statistical analysis, this study identified 34 polar metabolites whose abundances in placentas from spontaneous preterm births differed significantly from (and were generally lower than) those in healthy, full-term controls. We found that neither the spontaneous preterm delivery phenotype nor the HCA inflammatory response explained the observed differences in the placental metabolome. However, we identified two molecular subtypes of placentas from spontaneous preterm pregnancies using unsupervised clustering methods. We also found that early and late preterm samples had differing metabolomic profiles, suggesting distinct placental functions in early spontaneous preterm delivery. 

Only one other study has investigated the placental metabolome and its association with spontaneous preterm birth (Elshenawy et al., 2020[18]). This earlier study examined a relatively small clinical cohort of 19 spontaneous preterm and 19 term samples and focused on a broader range of metabolites than our analysis, including prostaglandins, sphingolipids, lysolipids, and acylcarnitines. In contrast, our study focuses on polar metabolites including amino acids, nucleic acids, and small organic acids that are involved in intracellular energy, metabolism and other biochemical processes. Consequently, some of the metabolites found to distinguish spontaneous preterm birth placentas in this study were not previously linked to this condition. Moreover, no other study has used metabolomic profiling to distinguish the two main clinical phenotypes of spontaneous preterm delivery. Here we show that while PTB and PPROM have different clinical presentations, they have similar placental metabolic signatures that are consistent with their similarities at the gene and protein levels (Faupel-Badger et al., 2011[19]; Karahoda et al., 2021[31]).

As building blocks of proteins, amino acids are one of the most important classes of nutrients necessary for placental and fetal growth. They also play a role in regulating cell metabolism, proliferation, growth, and differentiation. Apart from histidine, we found that the preterm placenta had reduced levels of both essential (leucine) and non-essential (alanine, glutamine, tyrosine) amino acids. Changes in amino acid abundance (mainly reductions in their concentrations) in preterm birth have previously been observed in maternal plasma, amniotic fluid, and cervicovaginal fluid (Carter et al., 2019[14]; Ghartey et al., 2015[20]; Graça et al., 2012[23]; Menon et al., 2014[41]; Romero et al., 2010[50]). Such reductions may be related to the role of amino acids in energy metabolism. Spontaneous preterm birth is characterized by enhanced oxidative stress in the placenta, which increases the tissue's metabolic rate, energy consumption, and oxygen requirement (Martin et al., 2018[40]). It also impairs the uptake of amino acids by the placenta (Araújo et al., 2013[4]), potentially altering their concentrations in the trophoblast. 

Some amino acids are precursors of hormones and neuroactive metabolites. For example, tryptophan has recently drawn significant attention because it may influence the fetal programming of mental health disorders (Bonnin and Levitt, 2011[9]; Goeden et al., 2013[21]). Placental intermediates that may be involved in this process include several potentially neuroactive metabolites such as serotonin, kynurenic acid (neuroprotective), and quinolinic acid (neurotoxic) (Bonnin et al., 2007[10]; Sedlmayr et al., 2014[55]). In this context, it is interesting that the flux of tryptophan along the kynurenine pathway differed significantly between spontaneous preterm and healthy term placentas: preterm placentas had reduced contents of kynurenine and kynurenic acid together with elevated levels of quinolinic acid. This shifted the quinolinic acid/kynurenic acid ratio in favor of the neurotoxic quinolinic acid, with potentially deleterious effects on the developing fetus. Interestingly, we have recently reported a similar shift in the quinolinic acid/kynurenic acid ratio in human placenta explants exposed to bacterial or viral infection (Abad et al., 2023[1]). Consistent with these findings, the umbilical blood of preterm fetuses was reported to have a high concentration of quinolinic acid (Manuelpillai et al., 2005[38]). Quinolinic acid is an essential substrate for the *de novo* synthesis of NAD^+^ (Broekhuizen et al., 2021[12]), so one might speculate that this increase in its concentration is an adaptive response to increase intracellular NAD^+^ regeneration in an environment of increased turnover and demand. However, the abundance of nicotinamide and the nicotinamide/quinolinic acid ratio in spontaneous preterm placentas were significantly lower than in healthy term placentas. Because nicotinamide is the end-product of cellular NAD^+^-consuming activities (Cercillieux et al., 2022[15]), this suggests that the quinolinic acid accumulates in the preterm placenta. Placental quinolinic acid homeostasis may thus be an important mechanistic component of the relationship between spontaneous preterm birth and an increased risk of neurodevelopmental, behavioral, and psychiatric disorders in adulthood (Nosarti et al., 2012[45]; Saigal and Doyle, 2008[51]).

Contrary to previous beliefs, recent research has revealed that preeclampsia and FGR are not the only conditions in which ischemia-induced placental bed abnormalities may occur (Romero et al., 2011[50]). Several studies have shown that these pathological abnormalities in preeclampsia, FGD, or IUGR are associated with altered placental expression of vasoactive molecules including arginine and purine metabolites, which are particularly important in the placental bed given its lack of neural innervation (Anderssohn et al., 2012[3]; Dai et al., 2020[17]; Holden et al., 1998[24]; Iriyama et al., 2015[26]; Many et al., 1996[39]; Salsoso et al., 2017[53]; Savvidou et al., 2003[54]). Our results provide new insights into the placental response in spontaneous preterm birth. First, we show that preterm placentas exhibit increased levels of arginine, a precursor for the synthesis of nitric oxide (NO) that is important in angiogenesis and tissue growth. Conversely, the levels of two methylated arginine analogs, asymmetric dimethylarginine (ADMA) and symmetric dimethylarginine (SDMA), were significantly reduced in spontaneous preterm placentas. ADMA is an endogenous inhibitor of the enzymatic synthesis of NO from arginine (Vallance et al., 1992[61]), so the increased arginine/ADMA ratio in preterm placentas indicates higher arginine bioavailability for NO production (Bode-Böger et al., 2007[8]). Elimination of ADMA depends on enzymatic metabolism by dimethylaminotransferase (DDAH), which is highly expressed in the placenta (Leiper et al., 1999[34]). Interestingly, the ADMA/SDMA ratio, which is a proxy for DDAH activity, was similar in term and preterm pregnancies, suggesting functionally unchanged enzymatic activity (Savvidou et al., 2003[54]). These findings differ markedly from those reported for FGR or IUGR pregnancies, in which the maternal plasma or placenta exhibit increased ADMA levels, impaired DDAH2 activity, and reduced NO production (Anderssohn et al., 2012[3]; Dai et al., 2020[17]; Holden et al., 1998[24]; Savvidou et al., 2003[54]). However, they are consistent with reports of low ADMA levels in cervicovaginal fluid from women with spontaneous preterm birth (Ghartey et al., 2015[20]), suggesting the involvement of different mechanisms in these two classes of pregnancies. 

The metabolomic analysis also revealed substantial changes in the levels of metabolites involved in purine breakdown. Specifically, spontaneous preterm placentas exhibited significantly increased levels of AMP, inosine, and guanosine together with reduced levels of adenine, adenosine, hypoxanthine, and urate. These results are inconsistent with previous reports stating that levels of adenosine and hypoxanthine (which both mediate local blood flow) are reduced in preeclampsia, probably because of local hypoxia and ischemia (Iriyama et al., 2015[26]; Many et al., 1996[39]; Salsoso et al., 2017[53]). Hypoxia is generally associated with increased degradation of ATP to AMP, which cannot be recycled due to a lack of oxygen and is therefore degraded into adenosine, hypoxanthine, and finally, uric acid (Maguire et al., 1992[37]). However, the adenosine/AMP ratio is reduced in spontaneous preterm placentas, indicating that the hydrolysis of AMP to adenosine is impaired, leading to an accumulation of AMP. Moreover, these placentas also showed evidence of increased deamination of adenosine to inosine together with significantly reduced phosphorolysis of inosine to hypoxanthine, leading to inosine accumulation. Although these findings seem counterintuitive, we speculate that despite the similar ischemic pathologies of the placental bed in spontaneous preterm birth and other ischemic placental diseases (e.g., preeclampsia and FGR), their differing clinical phenotypes may result from different adaptive hemodynamic mechanisms, as suggested by Romero et al. (2011[49]). These speculations await further experimental investigation.

The incidence of spontaneous preterm birth is increasing and this condition is unfortunately associated with adverse long-term effects on the newborn. In addition, the molecular and metabolic mechanisms involved in its etiology are poorly understood. The placenta is an attractive target for study in this context because of its critical position at the materno-fetal interface and its multiple functions during gestation (Staud and Karahoda, 2018[57]). Several placental metabolic pathways have already been implicated in fetal programming; in particular, placental tryptophan metabolism was identified as a potential mechanistic link between a disturbed prenatal environment and a predisposition to mental health disorders in adulthood (Goeden et al., 2013[21]). When faced with a compromised environment, the placenta can adapt at the cost of permanent changes to its own and fetal physiology (Burton et al., 2010[13]). However, finding diagnostic or therapeutic tools has proved challenging because the spontaneous preterm birth placenta has not previously been thoroughly characterized. 

Several transcriptomic studies have revealed placental physiological dysfunctions in spontaneous preterm birth that make the organ's adaptive mechanisms unable to maintain proper pregnancy and birth timing (Brockway et al., 2019[11]; Couture et al., 2023[16]; Lien et al., 2021[35]; Paquette et al., 2018[46], 2023[47]). By showing that a wide range of metabolites are similarly dysregulated in spontaneous preterm birth placenta, this work extends our understanding of these dysfunctions from the transcriptomic to the metabolomic levels. These metabolites may have only minor effects on the disease individually but collectively the changes in their concentrations could clarify the pathophysiology and complexity of spontaneous preterm delivery. Importantly, metabolomic profiling of newborns and young adults born preterm has revealed certain differences from children born to healthy full-term pregnancies (Nilsson et al., 2022[44]; Perrone et al., 2021[48]; Wilson et al., 2014[65]) that may reflect changes in placental programming at the metabolic level. As such, transcriptomic and metabolomic analyses of the placenta could provide valuable information to guide therapeutic interventions (including dietary ones) to ensure proper placental function and fetal programming.

Metabolomics techniques can be used to identify and study situations where multiple biological pathways are acting in concert and thus offer exciting possibilities for characterizing biological events associated with spontaneous preterm birth. The findings from this and other studies (Elshenawy et al., 2020[18]; Knijnenburg et al., 2019[33]; Paquette et al., 2018[46], 2023[47]) demonstrate the need for accurate phenotypic characterization of spontaneous preterm birth because they suggest that the physiological differences between early preterm delivery and healthy term delivery placentas are more pronounced than those seen for late preterm delivery placentas. Future study designs should take such information into account.

Our findings could also be hypothesis-generating, motivating further studies on the biological roles of individual metabolites. For example, we identified several placental pathways whose activity in spontaneous preterm birth differed from that seen in other ischemic placental diseases such as preeclampsia and FGR. In particular, further studies on the regulation of arginine and ADMA in the placenta are warranted to clarify their role in the etiology or adaptive mechanisms involved in spontaneous preterm birth. Similarly, little is known about purine metabolism in the placenta, and more research is needed to understand the function of purinergic signaling in the fetoplacental unit and its connection to spontaneous preterm birth. Finally, it would be interesting to assess the neurodevelopment of children born preterm in relation to the observed differences in placental quinolinic acid levels; studies along these lines are currently ongoing in our laboratory. 

It should be noted that our analysis could not distinguish metabolites whose dysregulation constitutes a causative pathogenic process from those whose changes result from an adaptive response to the condition. Further studies are therefore needed to determine the involvement of the metabolites discussed here in the etiology and pathology of spontaneous preterm birth. This would be particularly beneficial in identifying targets for therapeutic interventions. 

The primary strengths of this study are the inclusion of a large clinical cohort and the use of advanced multivariate methods to integrate detailed and well-defined demographic and clinical data for each patient. Medically induced preterm deliveries and pregnancy disorders (such as preeclampsia, IUGR, and gestational diabetes) were excluded from the cohort, so the detected metabolomic signature is characteristic only of spontaneous preterm births. To eliminate bias in patient classification, we also used unsupervised clustering to identify placental subgroups in our study population. Last but not least, we applied strict control protocols to ensure the reliability of the clinical, experimental, and analytical data.

A substantial limitation of this study is the lack of normal age-matched controls. This is an important drawback in preterm birth research, as previously discussed (Brockway et al., 2019[11]; Couture et al., 2023[16]; Paquette et al., 2023[47]). Consequently, the results should be interpreted with caution as some of the observed effects could be attributed to gestational-age-related changes as a confounding factor. Moreover, the mode of delivery varied significantly between term and preterm births, which may also have influenced the placental metabolome. While a previous study suggested that the mode of delivery does not alter placental metabolic characteristics (Visiedo et al., 2015[63]), there is no clear consensus on this matter. Lastly, our statistical analysis could not control for the effects of corticosteroid, antibiotic, and/or tocolytic treatment on the placental metabolomic profile. Further research is thus needed to determine the potential effects of these agents on the placental metabolome in spontaneous preterm birth. 

Taken together, our findings show that spontaneous preterm birth is associated with significant changes in the placental metabolome and support the use of “omics” to clarify the placenta's role in spontaneous preterm delivery. The metabolites discussed here may be involved in fetal developmental programming of cardiovascular, metabolic, and neurological disorders. As such, they could be valuable targets when designing biomarkers and therapeutic interventions to reduce the global health burden of spontaneous preterm delivery. 

## Notes

Eva Cifkova and Rona Karahoda contributed equally as first author.

Miroslav Lisa and Frantisek Staud (Department of Pharmacology and Toxicology, Faculty of Pharmacy in Hradec Kralove, Charles University, Akademika Heyrovskeho 1203/8, 50005, Hradec Kralove, Czech Republic; E-mail: frantisek.staud@faf.cuni.cz) contributed equally as corresponding author.

## Declaration

### Availability of data and materials

All data generated or analyzed during this study are included in this published article (and its additional files).

### Conflict of interest

The authors report no conflict of interest.

### Source of funding

This study was financially supported by the Czech Health Research Council (NU20-01-00264), Czech Science Foundation (22-13967S) and the project National Institute for Neurological Research (Programme EXCELES, ID Project No. LX22NPO5107) - funded by the European Union - Next Generation EU. EC and ML acknowledge financial support of the Faculty of Science, University of Hradec Kralove.

### Authors' contributions

EC - experimental design, metabolomic analysis, statistical analysis, manuscript writing; RK - sample preparation, statistical analysis, manuscript writing; JS - sample collection and characterization; CA - sample preparation, statistical analysis; MK - sample collection and characterization; ML - experimental design, metabolomic analysis, statistical analysis, funding; FS - hypothesis, experimental design, funding, manuscript writing. All authors reviewed and approved the manuscript. 

## Supplementary Material

Supplementary information

## Figures and Tables

**Table 1:**
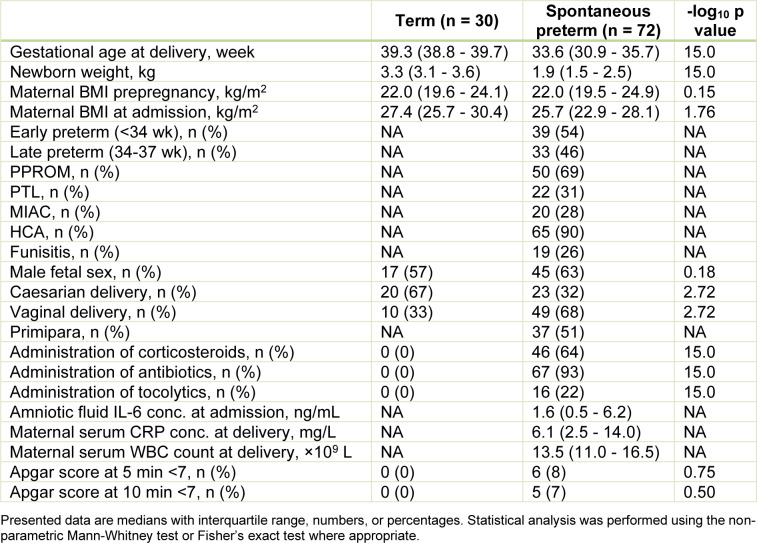
Clinical and demographic information on pregnancies included in the study
