# Supplementary material for: Metabolomic analysis of the human placenta reveals perturbations in amino acids, purine metabolites, and small organic acids in spontaneous preterm birth
Source: EXCLI J. 2024 Feb 13;23:264–82. doi: 10.17179/excli2023-6785 (PMC10938235; doi:10.17179/excli2023-6785)
Supplement: Supplementary information [file EXCLI-23-264-s-001.pdf]

EXCLI Journal 2024;23:264-282 – ISSN 1611-2156
Received: November 08, 2023, accepted: January 31, 2024, published: February 13, 2024

Supplementary information to:

Original article:

METABOLOMIC ANALYSIS OF THE HUMAN PLACENTA REVEALS PERTURBATIONS IN AMINO ACIDS, PURINE METABOLITES, AND SMALL ORGANIC ACIDS IN SPONTANEOUS PRETERM BIRTH

Eva Cifkova[1†], Rona Karahoda[2†], Jaroslav Stranik[3], Cilia Abad[2], Marian Kacerovsky[3], Miroslav Lisa[1*], Frantisek Staud[2*]

[1] Department of Chemistry, Faculty of Science, University of Hradec Kralove, Rokitanskeho 62, 50003, Hradec Kralove, Czech Republic
[2] Department of Pharmacology and Toxicology, Faculty of Pharmacy in Hradec Kralove, Charles University, Akademika Heyrovskeho 1203/8, 50005, Hradec Kralove, Czech Republic
[3] Department of Obstetrics and Gynecology, University Hospital Hradec Kralove, Sokolska 581, 50005, Hradec Kralove, Czech Republic

† These authors have contributed equally to this work.
* **Corresponding authors:** Miroslav Lisa, Department of Chemistry, Faculty of Science, University of Hradec Kralove, Rokitanskeho 62, 50003, Hradec Kralove, Czech Republic. E-mail: miroslav.lisa@uhk.cz;
Frantisek Staud, Department of Pharmacology and Toxicology, Faculty of Pharmacy in Hradec Kralove, Charles University, Akademika Heyrovskeho 1203/8, 50005, Hradec Kralove, Czech Republic. E-mail: frantisek.staud@faf.cuni.cz

https://dx.doi.org/10.17179/excli2023-6785

This is an Open Access article distributed under the terms of the Creative Commons Attribution License (http://creativecommons.org/licenses/by/4.0/).

EXCLI Journal 2024;23:264-282 – ISSN 1611-2156
Received: November 08, 2023, accepted: January 31, 2024, published: February 13, 2024

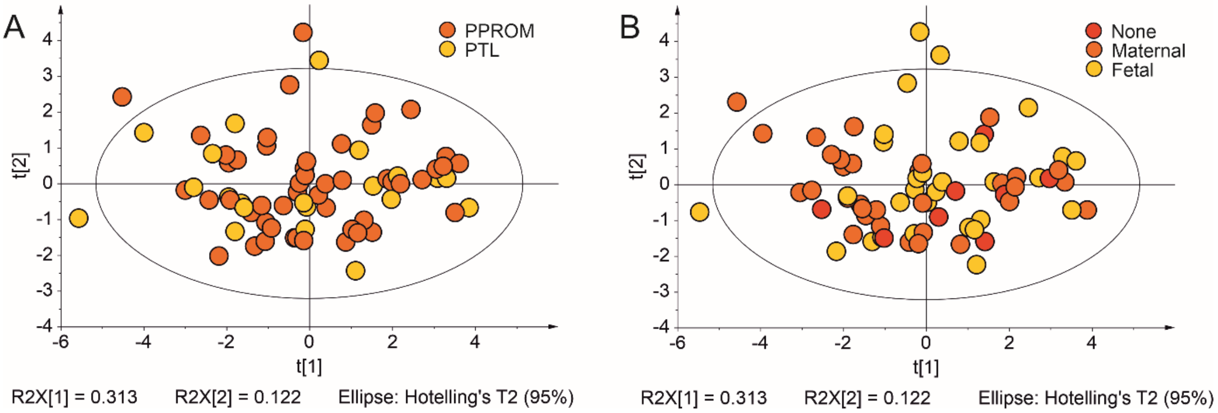

Supplementary Figure 1: Principal component analysis of metabolomic profiles for preterm birth placentas. Correlations between placentas associated with PPROM and PTL preterm birth phenotypes (**A**) and indicated HCA grades (**B**).

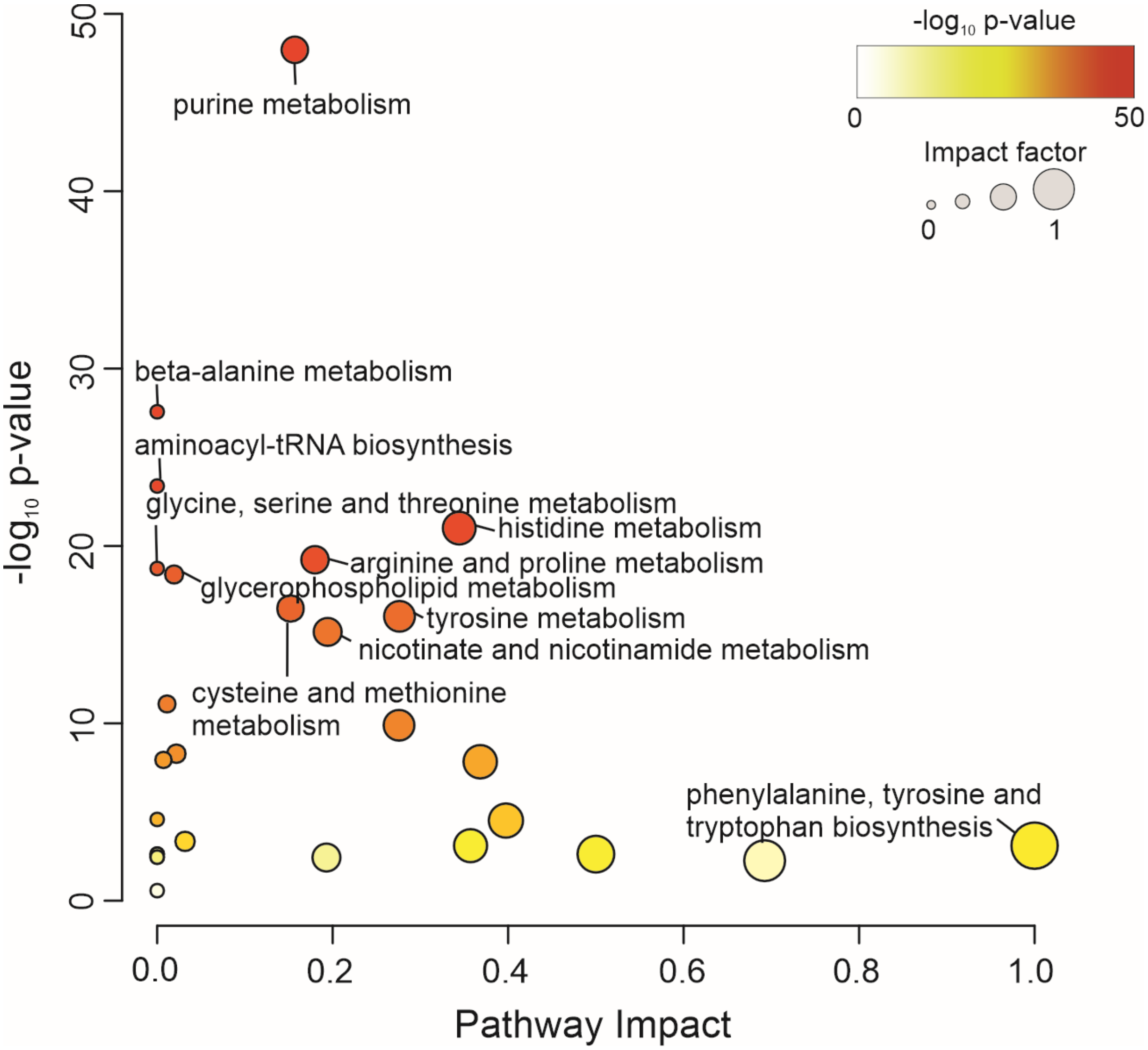

Supplementary Figure 2: Pathway enrichment analysis of significantly altered metabolites in spontaneous preterm birth placentas. The node color gradient indicates the negative log p-value and the node radius indicates the pathway impact score. The 10 most significantly affected metabolic pathways are identified by name.

EXCLI Journal 2024;23:264-282 – ISSN 1611-2156
Received: November 08, 2023, accepted: January 31, 2024, published: February 13, 2024

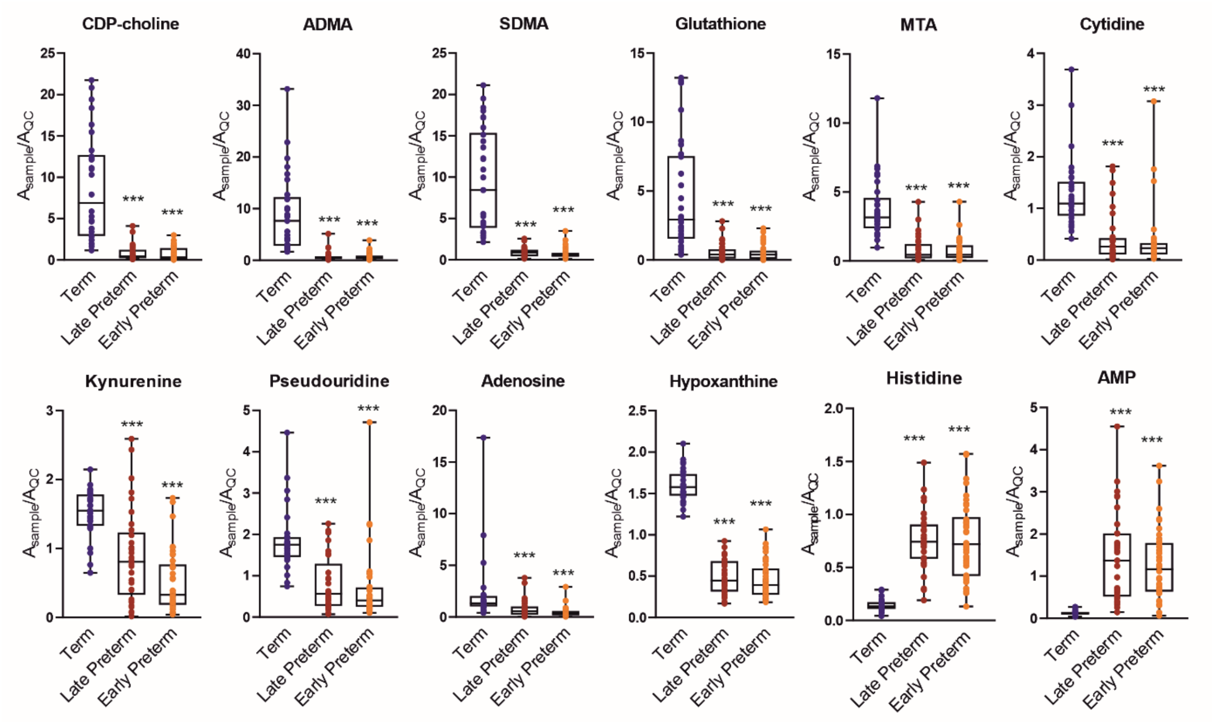

Supplementary Figure 3: Box plots of up- and down-regulated metabolites in early and late spontaneous preterm birth deliveries. Statistical analysis was performed using the nonparametric Mann-Whitney test, and the false discovery rate was performed using the Bonferroni and Benjamini-Hochberg corrections.

Supplementary Table 1: Clinical and demographic information on preterm pregnancies included in the study

	PTL (n = 22)	PPROM (n = 50)	$-\log_{10}$ p value
Gestational age at delivery, week	32.0 (27.2 - 34.1)	34.4 (31.9 - 36.0)	2.09
Newborn weight, kg	1.7 (1.0 - 2.2)	2.2 (1.7 - 2.6)	2.21
Maternal BMI prepregnancy, kg/m^2	21.0 (19.0 - 22.5)	23.1 (19.7 - 25.0)	1.12
Maternal BMI at admission, kg/m^2	23.1 (22.5 - 25.6)	26.5 (23.4 - 28.4)	1.48
Early preterm (<34 wk), n (%)	16 (73)	23 (46)	1.36
Late preterm (34-37 wk), n (%)	6 (27)	27 (54)	1.36
MIAC, n (%)	4 (18)	16 (32)	0.57
HCA, n (%)	22 (100)	43 (86)	1.03
Funisitis, n (%)	3 (14)	16 (32)	0.83
Male fetal sex, n (%)	14 (64)	31 (62)	0.00
Caesarian delivery, n (%)	8 (36)	15 (30)	0.22
Vaginal delivery, n (%)	14 (64)	35 (70)	0.22
Primipara, n (%)	11 (50)	26 (52)	0.00
Administration of corticosteroids, n (%)	16 (73)	30 (60)	0.37
Administration of antibiotics, n (%)	19 (86)	48 (96)	0.79
Administration of tocolytics, n (%)	11 (50)	5 (10)	3.40
Amniotic fluid IL-6 conc. at admission, ng/mL	4.7 (1.8 - 11.2)	1.0 (0.4 - 2.0)	2.74
Maternal serum CRP conc. at delivery, mg/L	7.5 (3.6 - 14.9)	4.8 (2.4 - 13.1)	0.49
Maternal serum WBC count at delivery, $\times 10^9$ L	13.8 (11.3 - 16.2)	13.4 (11.0 - 16.6)	0.01
Apgar score at 5 min <7, n (%)	5 (23)	1 (2)	2.05
Apgar score at 10 min <7, n (%)	3 (14)	2 (4)	0.79

Presented data are medians with interquartile range, numbers, or percentages. Statistical analysis was performed using the nonparametric Mann-Whitney test or Fisher's exact test where appropriate.

EXCLI Journal 2024;23:264-282 – ISSN 1611-2156
Received: November 08, 2023, accepted: January 31, 2024, published: February 13, 2024

Supplementary Table 2: Composition of the internal standard mixture used for metabolomic analysis

Internal standard	Concentration (ng/µL)
**GABA[D6]**	1050.29
**Aspartic acid[D3]**	1113.94
**Dopamine[D4]**	0.32
**Glutamic acid[D3]**	1050.29
**Kynurenic acid[D5]**	3.18
**Kynurenine[D4]**	3.18
**L-dopa[D3]**	2.55
**Leucine[D3]**	159.13
**Lysine[D4]**	286.44
**Serotonin[D4]**	0.64
**Epinephrine[D3]**	95.48
**3-methoxytyramine[D4]**	0.32

EXCLI Journal 2024;23:264-282 – ISSN 1611-2156
Received: November 08, 2023, accepted: January 31, 2024, published: February 13, 2024

Supplementary Table 3: UHPLC/MS method validation results for metabolomic analysis of human placenta samples

	Without matrix		With matrix		Linearity range [pmol/mL]	LOD [pmol/mL]	Accuracy [%]			Precision [%]			Extraction recovery [%]
							Intraday		Inter-day	Intraday		Inter-day	
	Slope	R^2	Slope	R^2			LL	HL	HL	LL	HL	HL	HL
GABA[D6]	0.1058	0.988	0.0064	0.982	6110-122211	109	100	101	95	9	4	9	78
Aspartic acid[D3]	53.250	0.997	0.002	0.980	48999–489993	8166	102	101	92	7	7	13	52
Dopamine[D4]	49.841	0.993	73.169	0.999	4–42435	1	100	95	98	9	3	7	51
Glutamic acid[D3]	7.871	0.998	0.070	0.996	1777–444231	223	101	100	95	5	3	8	78
Kynurenic acid[D5]	158.820	0.996	101.690	0.999	3–34351	1	102	92	97	9	4	9	48
Kynurenine[D4]	172.520	0.999	0.975	0.999	3–31430	1	106	105	91	15	11	16	63
L-dopa[D3]	24.067	0.989	33.966	0.996	3–33319	1	102	89	97	18	4	11	58
Leucine[D3]	0.469	0.993	N/A	N/A	99-9942	1657	101	102	93	10	8	13	71
Lysine[D4]	2.966	0.989	N/A	N/A	44-1776	1974	107	105	97	17	11	15	32
Serotonin[D4]	53.248	0.997	10.872	0.996	37-37012	2	102	97	94	8	6	9	31
Epinephrine[D3]	168.170	0.992	52.181	0.993	37-35821	1	99	95	97	7	5	6	74
3-methoxytyramine[D4]	87.138	0.995	8.166	0.999	39-38959	3	103	99	95	12	8	11	53

LL – low concentration level, HL – high concentration level

EXCLI Journal 2024;23:264-282 – ISSN 1611-2156
Received: November 08, 2023, accepted: January 31, 2024, published: February 13, 2024

Supplementary Table 4: Metabolite fold change and p-value after FDR corrections for identified metabolites

Rank	Metabolite	Negative log (p-value); significance after Bonferroni correction α = -log (0.05; 0.01; 0.001 / 43) = 2.9 (*); 3.6 (**); 4.6 (***)			Negative log (p-value); significance after Benjamini and Hochberg correction α = -log (RANK *0.05; 0.01; 0.001 / 43) = 1.39 (*); 2.11 (**); 3.16 (***)			Median fold change		
		Preterm vs. Term	Late Preterm vs. Term	Early Preterm vs. Term	Preterm vs. Term	Late Preterm vs. Term	Early Preterm vs. Term	Preterm vs. Term	Late Preterm vs. Term	Early Preterm vs. Term
41	5-Hydroxytryptophan	0.21	0.07	0.58	0.21	0.07	0.58	0.11	-0.03	0.27
12	Acetylcarnitine[a]	13.06	9.41	10.27	13.06	9.41	10.27	-0.38	-0.64	-0.70
18	Acetylneuraminate[a]	9.66	8.30	6.87	9.66	8.30	6.87	-0.51	-1.05	-1.03
26	Adenine[a]	5.72	3.22	5.59	5.72	3.22	5.59	-0.21	-0.31	-0.35
19	Adenosine	9.25	4.74	11.12	9.25	4.74	11.12	-0.72	-1.27	-2.05
1	ADMA	15.00	13.89	15.00	15.00	13.89	15.00	-0.93	-3.88	-4.04
3	AMP[a]	15.00	15.00	13.85	15.00	15.00	13.85	9.03	3.49	3.26
33	Arginine	1.98	1.98	1.15	1.98	1.98	1.15	0.23	0.31	0.29
40	Caffeine[a]	0.33	0.14	0.90	0.33	0.14	0.90	0.07	0.78	-0.20
25	Carnitine	6.40	5.00	5.11	6.40	5.00	5.11	-0.31	-0.52	-0.56
4	CDP-choline[a]	15.00	13.28	14.52	15.00	13.28	14.52	-0.95	-4.04	-4.46
38	CMP[a]	0.62	0.29	0.90	0.62	0.29	0.90	0.20	0.24	0.32
2	Creatine	15.00	11.53	15.00	15.00	11.53	15.00	-0.66	-1.47	-1.66
14	Cytidine	11.19	7.64	10.83	11.19	7.64	10.83	-0.76	-2.04	-2.25
21	GABA	8.46	5.67	8.17	8.46	5.67	8.17	-0.16	-0.24	-0.26
39	Glutamic acid	0.44	0.69	0.16	0.44	0.69	0.16	0.10	0.19	0.05
29	Glutamine	3.40	2.89	2.70	3.40	2.89	2.70	-0.26	-0.42	-0.45
11	Glutathione[a]	14.00	9.93	11.37	14.00	9.93	11.37	-0.87	-2.91	-2.96
42	GMP[a]	0.11	0.08	0.39	0.11	0.08	0.39	-0.11	0.19	-0.34
34	Guanosine	1.88	1.91	1.17	1.88	1.91	1.17	0.26	0.45	0.32
5	Histidine	15.00	15.00	15.00	15.00	15.00	15.00	4.54	2.48	2.44
6	Hypoxanthine	15.00	15.00	15.00	15.00	15.00	15.00	-0.73	-1.82	-2.01
24	Inosine	7.46	6.03	5.88	7.46	6.03	5.88	1.27	1.20	1.16
20	Kynurenic acid	8.79	7.28	6.69	8.79	7.28	6.69	-0.61	-1.12	-1.47
16	Kynurenine	9.94	4.80	11.31	9.94	4.80	11.31	-0.64	-0.94	-2.24
7	L-DOPA	15.00	12.06	11.24	15.00	12.06	11.24	-0.63	-1.35	-1.63
32	Leucine	2.61	1.13	3.05	2.61	1.13	3.05	-0.27	-0.16	-0.56

EXCLI Journal 2024;23:264-282 – ISSN 1611-2156
Received: November 08, 2023, accepted: January 31, 2024, published: February 13, 2024

Rank	Metabolite	Negative log (p-value); significance after Bonferroni correction α = -log (0.05; 0.01; 0.001 / 43) = 2.9 (*); 3.6 (**); 4.6 (***)			Negative log (p-value); significance after Benjamini and Hochberg correction α = -log (RANK *0.05; 0.01; 0.001 / 43) = 1.39 (*); 2.11 (**); 3.16 (***)			Median fold change		
		Preterm vs. Term	Late Preterm vs. Term	Early Preterm vs. Term	Preterm vs. Term	Late Preterm vs. Term	Early Preterm vs. Term	Preterm vs. Term	Late Preterm vs. Term	Early Preterm vs. Term
27	Metanephrine	5.29	3.28	5.02	5.29	3.28	5.02	-0.29	-0.47	-0.51
36	Methionine	0.87	1.07	0.50	0.87	1.07	0.50	-0.05	-0.09	-0.05
8	MTA[a]	15.00	11.53	13.46	15.00	11.53	13.46	-0.86	-2.85	-2.83
13	Nicotinamide	12.26	8.71	10.06	12.26	8.71	10.06	-0.47	-0.89	-0.94
23	Pantothenate[a]	7.87	4.53	7.63	7.87	4.53	7.63	-0.55	-0.94	-1.21
30	Paraxanthine	3.10	1.63	3.23	3.10	1.63	3.23	-0.55	-0.87	-1.51
43	Phenylalanine	0.00	0.48	0.31	0.00	0.48	0.31	-0.06	0.10	-0.34
17	Pseudouridine	9.91	6.03	9.26	9.91	6.03	9.26	-0.74	-1.63	-2.11
22	Quinolinic acid	8.44	5.89	8.00	8.44	5.89	8.00	1.26	0.98	1.33
15	SAH[a]	9.96	6.65	9.26	9.96	6.65	9.26	-0.39	-0.63	-0.91
9	SDMA	15.00	15.00	15.00	15.00	15.00	15.00	-0.92	-3.25	-3.92
31	Tryptophan	2.89	1.24	3.40	2.89	1.24	3.40	1.11	0.82	1.41
35	Tyramine	1.22	1.19	1.01	1.22	1.19	1.01	-0.10	-0.09	-0.16
28	Tyrosine	4.60	3.70	3.70	4.60	3.70	3.70	-0.25	-0.38	-0.51
10	Urate[a]	15.00	9.30	13.96	15.00	9.30	13.96	-0.45	-0.76	-0.92
37	Urocanate[a]	0.72	1.31	0.23	0.72	1.31	0.23	0.19	0.37	0.23

[a] Annotated metabolites without identical standard

EXCLI Journal 2024;23:264-282 – ISSN 1611-2156
Received: November 08, 2023, accepted: January 31, 2024, published: February 13, 2024

Supplementary Table 5: Spearman's correlation analysis

Metabolite	GA		CRP		IL6		Fetal weight		BMI at delivery		WBC at delivery	
	r	p-value	r	p-value	r	p-value	r	p-value	r	p-value	r	p-value
5-Hydroxytryptophan	-0.245	0.039	0.144	0.235	0.192	0.111	-0.292	0.013	-0.104	0.388	0.289	0.015
Acetylcarnitine[a]	0.087	0.469	0.071	0.559	-0.001	0.995	0.083	0.489	0.238	0.046	0.035	0.775
Acetylneuraminate[a]	-0.053	0.660	0.194	0.108	0.067	0.582	0.020	0.870	0.285	0.016	0.100	0.411
Adenine[a]	0.064	0.594	0.157	0.196	0.013	0.918	0.038	0.751	0.006	0.960	0.213	0.077
Adenosine	0.304	0.010	0.014	0.909	0.007	0.954	0.295	0.013	0.054	0.653	-0.036	0.767
ADMA	0.185	0.122	0.086	0.479	-0.091	0.453	0.145	0.229	0.042	0.725	-0.057	0.641
AMP[a]	0.067	0.576	0.137	0.259	-0.024	0.841	0.090	0.457	0.194	0.106	-0.052	0.670
Arginine	0.152	0.205	-0.043	0.722	-0.121	0.320	0.143	0.234	-0.065	0.592	-0.064	0.596
Caffeine[a]	0.234	0.050	-0.137	0.258	-0.282	0.018	0.210	0.079	0.055	0.652	-0.255	0.033
Carnitine	0.109	0.366	0.052	0.671	-0.067	0.582	0.082	0.498	0.182	0.129	-0.015	0.905
CDP-choline[a]	0.064	0.593	0.104	0.391	-0.002	0.987	0.090	0.456	0.197	0.100	0.029	0.812
CMP[a]	-0.037	0.761	0.200	0.096	0.053	0.662	-0.016	0.893	0.187	0.119	0.079	0.517
Creatine	0.149	0.214	0.035	0.776	0.023	0.853	0.189	0.114	0.055	0.650	-0.101	0.404
Cytidine	0.165	0.168	-0.032	0.792	0.130	0.284	0.138	0.251	-0.028	0.820	-0.033	0.786
GABA	0.163	0.173	0.136	0.262	-0.015	0.905	0.064	0.593	0.022	0.854	-0.124	0.309
Glutamic acid	0.083	0.491	0.053	0.664	-0.093	0.444	0.115	0.340	0.144	0.232	-0.032	0.793
Glutamine	-0.039	0.750	0.042	0.728	0.208	0.084	0.036	0.766	-0.089	0.461	-0.022	0.859
Glutathione[a]	-0.107	0.373	0.264	0.027	0.099	0.415	-0.115	0.339	0.019	0.877	-0.009	0.943
GMP[a]	0.201	0.093	0.079	0.516	-0.008	0.947	0.153	0.203	-0.024	0.845	-0.097	0.425
Guanosine	0.261	0.028	0.080	0.512	0.091	0.454	0.271	0.022	0.106	0.381	0.112	0.356
Histidine	0.029	0.809	0.091	0.454	-0.117	0.335	0.025	0.838	0.008	0.946	0.008	0.948
Hypoxanthine	0.201	0.093	-0.026	0.829	-0.040	0.743	0.182	0.129	0.027	0.825	-0.007	0.956
Inosine	0.117	0.333	0.091	0.454	0.042	0.730	0.130	0.278	0.078	0.519	0.081	0.506
Kynurenic acid	0.102	0.395	-0.123	0.309	0.182	0.132	0.120	0.320	0.088	0.466	0.097	0.425
Kynurenine	0.411	0.000	0.123	0.311	-0.072	0.554	0.380	0.001	0.067	0.579	-0.043	0.722

Metabolite	GA		CRP		IL6		Fetal weight		BMI at delivery		WBC at delivery	
	r	p-value	r	p-value	r	p-value	r	p-value	r	p-value	r	p-value
L-DOPA	0.076	0.526	-0.135	0.265	0.014	0.909	0.118	0.327	0.016	0.894	-0.038	0.753
Leucine	0.197	0.100	0.157	0.195	0.016	0.896	0.176	0.142	0.095	0.432	-0.071	0.559
Metanephrine	0.214	0.074	-0.050	0.681	-0.020	0.867	0.250	0.035	0.113	0.348	0.013	0.912
Methionine	-0.069	0.570	0.088	0.467	0.050	0.684	-0.078	0.519	-0.138	0.251	0.087	0.475
MTA[a]	0.007	0.954	0.128	0.293	-0.006	0.964	0.073	0.545	0.315	0.007	0.035	0.777
Nicotinamide	0.220	0.066	0.133	0.272	0.030	0.806	0.147	0.222	0.110	0.360	0.029	0.809
Pantothenate[a]	0.148	0.219	0.072	0.553	-0.084	0.491	0.167	0.164	0.147	0.221	-0.164	0.174
Paraxanthine	0.134	0.267	0.018	0.884	-0.099	0.414	0.060	0.617	-0.087	0.470	-0.079	0.515
Phenylalanine	0.267	0.025	-0.145	0.232	0.120	0.322	0.239	0.045	-0.049	0.684	0.015	0.900
Pseudouridine	0.227	0.057	-0.092	0.450	0.008	0.945	0.224	0.060	0.063	0.602	0.004	0.974
Quinolinic acid	-0.149	0.215	0.158	0.190	0.079	0.515	-0.221	0.064	0.197	0.099	0.124	0.306
SAH[a]	0.300	0.011	0.188	0.119	-0.046	0.708	0.266	0.025	0.089	0.462	-0.045	0.712
SDMA	0.270	0.023	-0.021	0.866	-0.099	0.417	0.310	0.009	0.217	0.069	-0.063	0.604
Tryptophan	-0.206	0.084	-0.069	0.570	0.201	0.095	-0.224	0.060	-0.001	0.992	0.074	0.542
Tyramine	0.015	0.900	-0.026	0.829	0.018	0.881	-0.017	0.890	-0.140	0.243	-0.063	0.605
Tyrosine	0.123	0.309	-0.155	0.202	0.006	0.960	0.095	0.429	-0.255	0.032	0.064	0.599
Urate[a]	0.269	0.023	0.205	0.089	-0.092	0.449	0.286	0.016	0.309	0.009	0.006	0.963
Urocanate[a]	0.255	0.032	-0.011	0.929	0.089	0.466	0.169	0.159	-0.264	0.026	0.200	0.096

[a] Annotated metabolites without identical standard
